# Genome-Wide Analysis of the Aquaporin Gene Family Reveals the Role of *NnPIP2-7* in Conferring Salt Tolerance in Lotus (*Nelumbo nucifera* Gaertn.)

**DOI:** 10.3390/plants15142186

**Published:** 2026-07-16

**Authors:** Kebin Mu, Wei Sheng, Lingyun Wang, Mingxing Zhu, Lin Shi, Zhaisheng Zheng, Liuyin Xie, Xiaoyang Chen, Yingchun Xu

**Affiliations:** 1 Jinhua Laboratory of Biological Seed Industry and Modern Agricultural Machinery, Jinhua Academy of Agricultural Sciences (Zhejiang Institute of Agricultural Machinery), Jinhua 321000, China; mukebin930903@163.com (K.M.); sw619977@163.com (W.S.); wly1984180099@163.com (L.W.); 15158802360@163.com (M.Z.); shiyibeinan@163.com (L.S.); zzs165@163.com (Z.Z.); 2 Provincial Key Laboratory of Characteristic Aquatic Vegetable Breeding and Cultivation, Jinhua Academy of Agricultural Sciences (Zhejiang Institute of Agricultural Machinery), Jinhua 321000, China; 3 College of Horticulture, Nanjing Agricultural University, Nanjing 210095, China; 15189786134@163.com; 4 Seed Management Station of Zhejiang Province, Hangzhou 310020, China

**Keywords:** *Nelumbo nucifera*, aquaporin, genome-wide identification, salt tolerance, *NnPIP2-7*, expression profiling

## Abstract

Salt stress represents a major constraint on the productivity of lotus (*Nelumbo nucifera*), an aquatic crop of significant ornamental, nutritional, and medicinal value. Although aquaporins (AQPs) are key regulators of plant water and solute homeostasis, the genomic organization and stress-responsive functions of this gene family in lotus remain largely uncharacterized. In this study, a total of 32 *NnAQP* genes were identified in the lotus genome and classified into five subfamilies. Segmental duplication was identified as the primary driver of family expansion under strong purifying selection. Expression profiling revealed broad transcriptional activity across various tissues, and qRT-PCR analysis demonstrated that *NnPIP2-7* was significantly and sustainedly induced under salt stress. Subcellular localization confirmed that NnPIP2-7 is targeted to the plasma membrane. Heterologous overexpression of *NnPIP2-7* in *Arabidopsis thaliana* significantly improved seed germination and primary root elongation under saline conditions. Crucially, transgenic plants exhibited enhanced osmotic adjustment and elevated antioxidant capacity, resulting in reduced oxidative damage compared to wild-type plants. These findings establish the evolutionary trajectory of the lotus *AQP* family and demonstrate the core regulatory role of *NnPIP2-7* in plant salt stress adaptation.

## 1. Introduction

Plants are sessile organisms that are continuously exposed to fluctuating environmental conditions, including various biotic and abiotic stresses that constrain vegetative growth and reproductive development [1]. Among these, salt stress is a major abiotic factor limiting plant productivity and causing substantial yield losses worldwide [2,3]. Saline soils account for approximately 6% of the global land area, affecting more than 800 million hectares [4,5]. Salt stress disrupts multiple physiological processes in plants, including seed germination, seedling establishment, vegetative growth, and reproductive development, with particularly pronounced effects on flowering and fruit maturation [6,7]. To cope with osmotic and ionic disturbances caused by salinity, plants rely on a suite of adaptive mechanisms, among which AQPs play a central role in regulating water and solute homeostasis.

AQPs belong to the evolutionarily conserved major intrinsic protein (MIP) family. They are integral membrane channel proteins characterized by six transmembrane α-helical domains and two signature selectivity motifs: the asparagine–proline–alanine (NPA) domain and the aromatic arginine (ar/R) selectivity filter [8,9]. The activity and transcript levels of AQPs are regulated by diverse environmental factors, including osmotic gradients, ionic imbalances, temperature extremes, hypoxia, and heavy metal stress [10,11,12,13,14]. In seed plants, AQPs are further classified into five subfamilies: plasma membrane intrinsic proteins (PIPs), tonoplast intrinsic proteins (TIPs), nodulin-26-like intrinsic proteins (NIPs), small basic intrinsic proteins (SIPs), and unrecognized X-intrinsic proteins (XIPs) [15]. Two additional subfamilies, GlpF-like Intrinsic Proteins (GIPs) and Hybrid Intrinsic Proteins (HIPs), have been identified in the bryophyte *Physcomitrella patens* and the lycophyte *Selaginella moellendorffii*, respectively [16,17]. PIPs, localized to the plasma membrane, are further divided into PIP1 and PIP2 subgroups, which differ primarily in the lengths of their N- and C-terminal domains [18,19]. TIPs display broader subcellular distribution, having been detected in the vacuolar membrane, chloroplasts, plasma membrane, endoplasmic reticulum, and mitochondria [20,21,22,23]. NIPs are exclusive to plants and can be subdivided into three groups (NIP I–III) based on the amino acid composition of the ar/R selectivity filter, which determines substrate specificity for molecules such as silicon, boron, and arsenic [24,25,26]. SIPs possess a notably short N-terminal region and remain the least characterized subfamily in terms of intracellular localization [27,28]. XIPs, initially discovered in mosses, are absent in monocots and Brassicaceae but are present in many eudicots, where they localize to the plasma membrane and endomembrane system [17,29,30,31,32,33].

Since the initial discovery of the aquaporin CHIP28, now designated AQP1, homologous AQP isoforms have been identified and characterized across diverse plant species [34,35]. Advances in genome sequencing have enabled comprehensive genome-wide analyses of AQP gene families, revealing 35 members in *Arabidopsis* [28], 35 in *Eutrema salsugineum* [36], 33 in *Oryza sativa* [37], 68 in *Gossypium hirsutum* [38], and 24 in *Spirodela polyrhiza* [39]. Although AQPs were initially characterized as water-transporting channels, subsequent studies have demonstrated that they also mediate the bidirectional movement of reactive oxygen species (ROS) like H_2_O_2_, respiratory gases such as CO_2_ and O_2_, and other small solutes [40,41,42,43,44]. This functional versatility positions AQPs as key regulators of cellular homeostasis in response to environmental stresses. In the context of salt tolerance, several AQPs have been functionally validated in dicotyledonous crops, providing a highly relevant comparative context for *Nelumbo nucifera*. For instance, overexpression of the wild soybean (*Glycine soja*) aquaporin *GsPIP2;1* significantly increases salt tolerance in transgenic plants by regulating osmotic balance and reducing ion toxicity [45]. Similarly, in tomato (*Solanum lycopersicum*), the tonoplast aquaporin *SlTIP2;2* enhances salinity tolerance by facilitating intracellular osmotic adjustment and maintaining reactive oxygen species homeostasis [46]. Moreover, salt stress-induced changes in root hydraulic conductivity have been attributed largely to altered AQP activity [13]. Despite extensive characterization of AQP families in model and crop species, the *AQP* gene family in lotus (*Nelumbo nucifera*) and its regulatory roles under salt stress have not been systematically investigated.

Lotus (*Nelumbo nucifera* Gaertn.) is an economically important perennial aquatic plant belonging to the monotypic genus *Nelumbo*, which comprises two extant species: *N. nucifera* (Asian lotus) and *N. lutea* (American lotus). Based on agricultural utility, cultivated lotus is categorized into three types: flower-lotus, rhizome-lotus, and seed-lotus [47,48]. In recent years, the increasing need to utilize coastal saline soils for agricultural production in southeastern China, coupled with worsening soil salinization caused by continuous cropping obstacles in protected cultivation systems, has created an urgent need to enhance salt tolerance in lotus. This enhancement would facilitate the productive utilization of coastal saline-alkali lands and enable the expansion of lotus cultivation into marginal areas [49]. However, the commonly cultivated lotus varieties show high sensitivity to saline conditions [50,51]. Elucidating the molecular mechanisms of salt tolerance is therefore essential for developing salt-tolerant lotus varieties. While physiological responses and several stress-related genes, such as CIPKs and ERF transcription factors [50,52], have been characterized in lotus, the aquaporin gene family—which is central to water and solute transport under abiotic stress—has not been systematically investigated in this species.

In this study, we performed a genome-wide identification and systematic characterization of the *AQP* gene family in *N. nucifera*. We analyzed phylogenetic relationships, gene structures, conserved motifs, cis-regulatory elements, and synteny relationships of the *NnAQP* genes. Expression patterns were examined across different tissues and under salt stress treatment. Notably, our expression profiling revealed that *NnPIP2-7* was the most rapidly and intensely induced member under salt stress, exhibiting an early 10-fold up-regulation. Given this unique transcriptional responsiveness and its strict plasma membrane localization, we hypothesized that *NnPIP2-7* serves as a primary regulatory hub for osmotic adjustment in lotus. To validate this hypothesis and bridge the gap between genomic identification and biological function, we functionally validated the role of *NnPIP2-7* in conferring salt tolerance through heterologous expression and phenotypic analysis. These results provide a comprehensive genomic framework for the *NnAQP* family and offer new insights into the molecular basis of salt tolerance in lotus, with potential applications in the breeding of stress-resilient cultivars.

## 2. Results

### 2.1. Identification and Characterization of AQPs in Lotus

Through homology-based searches employing AQP amino acid sequences from *Arabidopsis thaliana* and *Oryza sativa*, along with XIP subfamily sequences from *Lotus japonicus* and *Solanum lycopersicum*, we systematically identified 32 non-redundant, full-length *NnAQP* members within the *N. nucifera* genome; these 32 *NnAQP* genes are unevenly distributed across the eight chromosomes of lotus (Figure S1). Table 1 lists the characteristic parameters of all predicted AQP members including protein length, molecular weight, theoretical pI, and grand average of hydropathicity (GRAVY). The identified AQP proteins in *N. nucifera* ranged from 233 amino acid residues (NnSIP2-3) to 407 amino acid residues (NnPIP2-2). Molecular weights ranged from 25.12 kDa (NnTIP2-1) to 44.18 kDa (NnPIP2-2). Theoretical pI ranged from 5.35 (NnTIP2-2) to 9.88 (NnSIP2-1). The GRAVY values of all NnAQPs were positive (ranging from 0.221 to 1.012), indicating that they were all hydrophobic.

### 2.2. Phylogenetic Analysis of the Lotus AQP Gene Family

**Figure 1:**
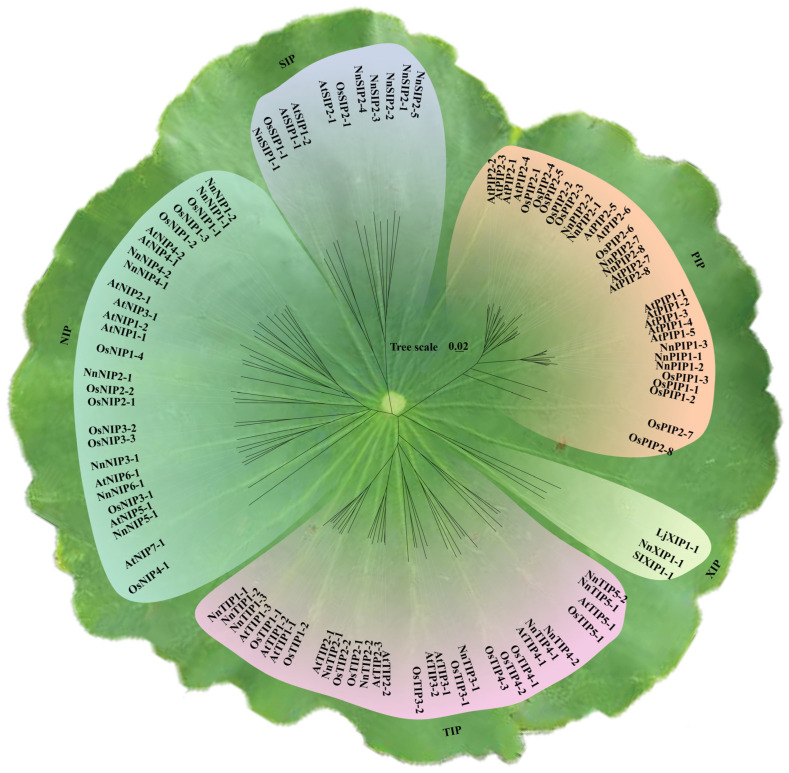
Phylogenetic analysis of the AQP gene family in *Nelumbo nucifera* and other representative plant species. The unrooted neighbor-joining (NJ) phylogenetic tree was constructed using AQP protein sequences from *N. nucifera* (Nn), *Arabidopsis thaliana* (At), *Oryza sativa* (Os), *Lotus japonicus* (Lj), and *Solanum lycopersicum* (Sl), with 1000 bootstrap replicates. The five AQP subfamilies (PIPs, NIPs, TIPs, SIPs, and XIPs) are highlighted with different background colors. The scale bar indicates the evolutionary distance in terms of amino acid substitutions per site.

To investigate the classification and evolutionary relationships of AQP proteins in *N. nucifera*, a phylogenetic tree was constructed using the Neighbor-Joining (NJ) method (Figure 1). This analysis was based on sequence similarity comparisons of AQP proteins among *N. nucifera*, *Arabidopsis thaliana*, *Oryza sativa*, *L. japonicus*, and *S. lycopersicum*. The *N. nucifera* AQPs were subsequently named based on their grouping with well-characterized AQPs from these species. Analysis of their affinities revealed five distinct evolutionary branches (PIPs, NIPs, TIPs, SIPs, and XIPs), each representing a major AQP subfamily. This classification into five subfamilies is consistent with the established AQP classification observed in other angiosperms. The analysis identified seven PIPs, eight NIPs, ten TIPs, six SIPs, and one XIP. Within the PIP subfamily, the seven members segregated into two groups: with three members in PIP1 and four members in PIP2. Similarly, the SIPs formed two subgroups: comprising one member in SIP1 and five members in SIP2. The TIP subfamily comprised five groups (TIP1 to TIP5), with TIP1 containing three members, TIP2, TIP4, and TIP5 each containing two members, and TIP3 containing one member. NIPs were divided into six subgroups: NIP1 and NIP4 had two members, while NIP2, NIP3, NIP5, and NIP6 each had one member. Finally, the XIP subfamily consisted of a single group.

### 2.3. Conserved Motifs, Domains, Gene Structure and Functional Residues of AQP Family Genes in Lotus

**Figure 2:**
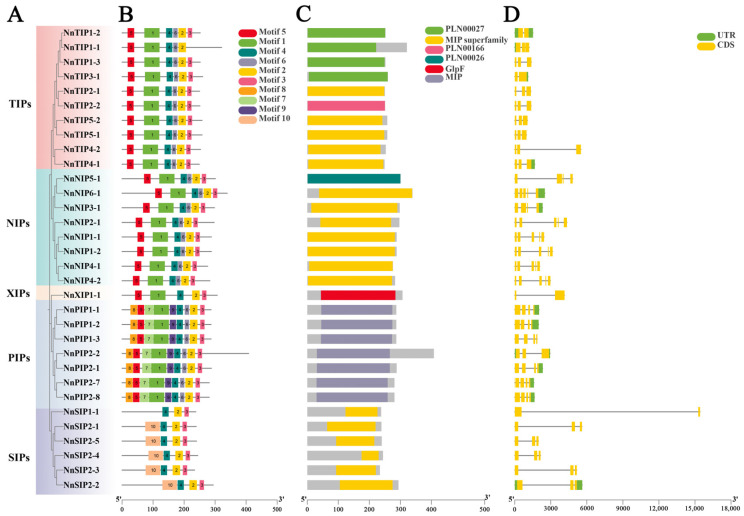
Conserved motifs, domains, gene structure, and functional residues of AQPs family genes in Lotus. (**A**) Phylogenetic tree of 32 NnAQP proteins, depicting the phylogenetic relationships among subfamily members with colored background shading indicating subfamily grouping (TIPs, NIPs, XIPs, PIPs, and SIPs). (**B**) Distribution of 10 conserved motifs (motif 1–10) identified by MEME analysis; each motif is represented by a distinct color. (**C**) Conserved domain architecture of NnAQP proteins, including MIP superfamily, PLN00027, PLN00166, PLN00026, GlpF, and MIP domains. The grey boxes indicate regions where no specific conserved domains were identified (**D**) Exon–intron gene structures of *NnAQP* genes; yellow boxes represent coding sequences (CDS), green boxes represent untranslated regions (UTRs), and black lines represent introns.

Protein motifs represent conserved sequence patterns or regions that are often associated with essential functional or structural roles within active proteins [53]. In this study, the motifs of 32 NnAQP family members were analyzed by the MEME online program, and 10 conserved motifs, designated as motif 1 to motif 10, were identified (Figure 2B). Notably, the SIP subfamily contains only 3–4 motifs, whereas all other subfamily members possess 5–9 motifs. Despite this motif reduction, all NnAQP members strictly retain Motif 2, 3, and 4, suggesting that these constitute the indispensable structural core determining the function of AQP family genes. Conserved domain analysis of AQP family demonstrated that all members of the NnAQP contain MIP domains, supporting their identification as putative aquaporins (Figure 2C). Variations in gene structure resulting from distinct exon and intron combinations provide valuable insights into the functional diversity of genes and the evolution of genomes [54]. To explore the structural diversity of *NnAQP* genes, the intron–exon structures of the lotus AQPs were analyzed (Figure 2D). The exon count within the *NnAQP* gene family displayed a range of 2–5, with a predominant three-exon configuration observed in 37.5% of members (12 out of 32 genes). Notably, *NnTIP3-1*, *NnTIP1-2*, *NnXIP1-1*, and *NnSIP1-1* exhibited the simplest genomic organization, each containing merely two exons, marking the minimal exon configuration observed. Phylogenetic clustering demonstrated high structural conservation among evolutionarily related members (Figure 2A). Specifically, the conserved five-exon architecture characterized the majority of NIP subfamily members, with two exceptions: *NnNIP3-1* and *NnNIP5-1* diverged from this pattern through secondary loss of one exon, resulting in a four-exon structure. This subfamily-specific conservation pattern suggests evolutionary maintenance of ancestral exon–intron organization within major phylogenetic subgroups.

Analysis of conserved functional residues revealed that most NnAQPs retained the characteristic dual NPA motifs. However, specific substitutions in these motifs, which are known to significantly affect channel permeability and substrate specificity, were observed in several subfamilies. For instance, the canonical NPA sequences were substituted by NPS and NPV in several NIP members, SPA and NPV in TIPs, and NPV in the XIP member. Notably, the SIP subfamily exhibited the greatest sequence variation, with the typical NPA motifs being replaced by highly diverse variants including NPT, NPL, NPI, NPF, and NSA (Figure S2, Table S1). To further validate their substrate specificity and evolutionary conservation, these residues were aligned with well-characterized AQPs from *Arabidopsis*, rice, *Lotus japonicus*, and *Solanum lycopersicum* (Figure S2). The ar/R selectivity filter was strictly conserved within the PIP subfamily (F/H/T/R) across all compared species, strongly supporting their functional roles as typical water channels. Likewise, Froger’s positions were generally conserved within each subfamily and aligned closely with their orthologs in the reference species, although minor variations were detected in several members (Figure S2, Table S1).

### 2.4. Cis-Regulatory Element Analysis in the Promoters of AQP Genes in Lotus

**Figure 3:**
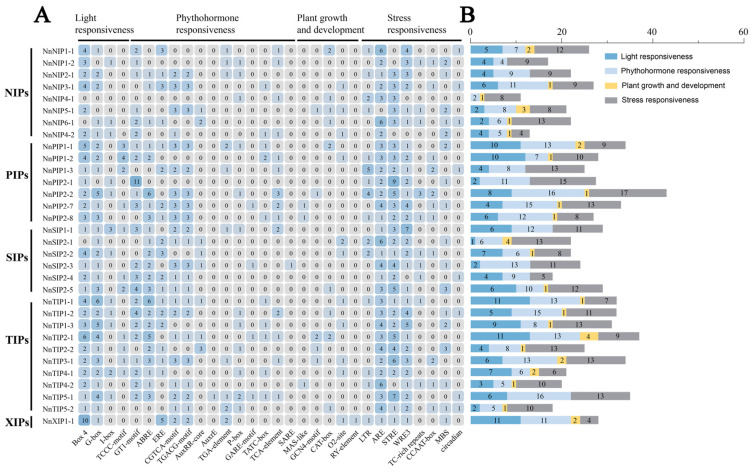
Analysis of cis-acting regulatory elements (CREs) in the promoter regions of *NnAQP* genes. (**A**) Heatmap showing the distribution and frequency of 30 CRE types identified within the 2000 bp upstream sequences of the 32 *NnAQP* genes. CREs are categorized into four functional groups: light responsiveness, phytohormone responsiveness, plant growth and development, and stress responsiveness. Color intensity reflects the number of each CRE type per gene, as indicated by the scale bar. The numbers inside the heatmap cells represent the exact count of the respective *cis*-acting regulatory elements. (**B**) Stacked bar chart summarizing the total count of CREs in each functional category for individual *NnAQP* genes.

Cis-regulatory elements (CREs) mediate gene expression regulation through interaction with trans-acting factors. To elucidate the transcriptional control mechanisms governing *NnAQP* genes, we systematically characterized CREs through computational analysis of 2000 bp promoter sequences upstream of each coding region using the PlantCARE online website (Figure 3). Bioinformatic analysis revealed 30 distinct types of CREs across four functional categories within the promoter regions of *NnAQP* genes, corresponding to transcriptional regulation of light responsiveness, plant growth and development, stress responsiveness and phytohormone responsiveness. Of the 32 gene promoters screened, STRE, ARE, Box 4, WRE3, GT1-motif, and ABRE were the six most commonly identified cis-elements with frequencies of 90, 90, 79, 64, 56, and 52, respectively. Furthermore, STRE, ARE, and WRE3 are all associated with drought and salt stress responses, while GT1-motif and ABRE are involved in phytohormone responses. These results suggest that most *NnAQPs* may play important roles in adaptation to environmental stresses in *N. nucifera*.

### 2.5. Analysis of Intraspecific and Interspecific Collinearity in the AQP Family Genes

**Figure 4:**
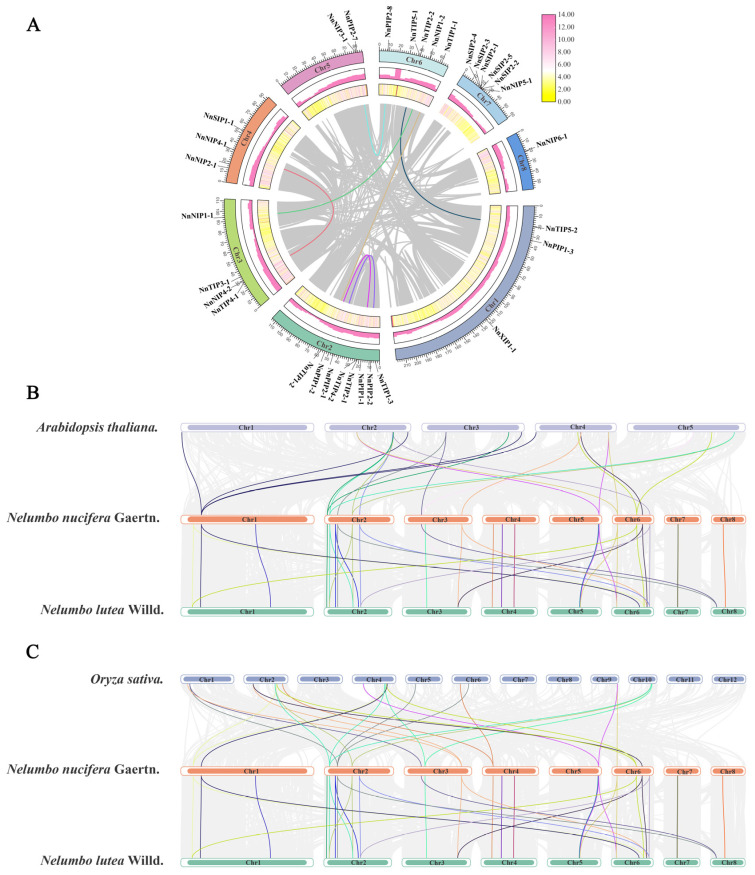
Intraspecific collinearity and interspecific synteny analysis of *NnAQP* genes. (**A**) Chromosomal distribution and intraspecific collinearity of *NnAQP* genes within the *Nelumbo nucifera* genome. The colored lines connect the segmentally duplicated *NnAQP* gene pairs, while the background gray lines represent genome-wide collinear blocks. (**B**) Interspecific synteny analysis among *Arabidopsis thaliana*, *N. nucifera*, and *Nelumbo lutea*. (**C**) Interspecific synteny analysis among *Oryza sativa*, *N. nucifera*, and *N. lutea*. In both (**B**,**C**), the colored lines highlight the specific orthologous AQP gene pairs across the species, whereas the gray lines in the background indicate the genome-wide syntenic blocks.

Analysis of intra-genomic collinearity revealed that seven *N. nucifera AQP* gene pairs, including *NnNIP1-1*/*NnNIP1-2*, *NnNIP4-1*/*NnNIP4-2*, *NnPIP1-1*/*NnPIP1-2*, *NnPIP2-1*/*NnPIP2-2*, *NnPIP2-7*/*NnPIP2-8*, *NnTIP1-1*/*NnTIP1-2* and *NnTIP5-1*/*NnTIP5-2* on 6 chromosomes in *N. nucifera*, all displayed segmental duplication events (Figure 4A, Table S2). These results suggest that segmental duplication events have contributed to the lotus *AQP* gene family expansion, which may be the primary driving force of expansion. Synteny analysis was used to evaluate the evolutionary process of *N. nucifera*, *N. lutea*, *A. thaliana* and *O. sativa* genes. Based on the syntenic results, 26, 14 and 13 of *NnAQP* genes in *N. nucifera* were syntenic with genes in *N. lutea*, *A. thaliana* and *O. sativa*, respectively, resulting in 42, 23 and 24 orthologous gene pairs (Figure 4B,C), indicating that the gene family is highly conserved in lotus. The substitution rate (non-synonymous/synonymous, Ka/Ks) is an effective criterion to evaluate the selective pressure during gene duplication [52]. Estimation of selective pressure revealed Ka/Ks < 1 for all segmentally duplicated *NnAQP* gene pairs, suggesting that purifying selection has shaped their evolution. In addition, the divergence time for these *NnAQP* duplication gene pairs were estimated based on Ks values, and it ranged from 39.43 to 53.74 million years ago (Mya) (Table S2).

### 2.6. Secondary and Tertiary Structure Analysis of NnAQP Proteins

**Figure 5:**
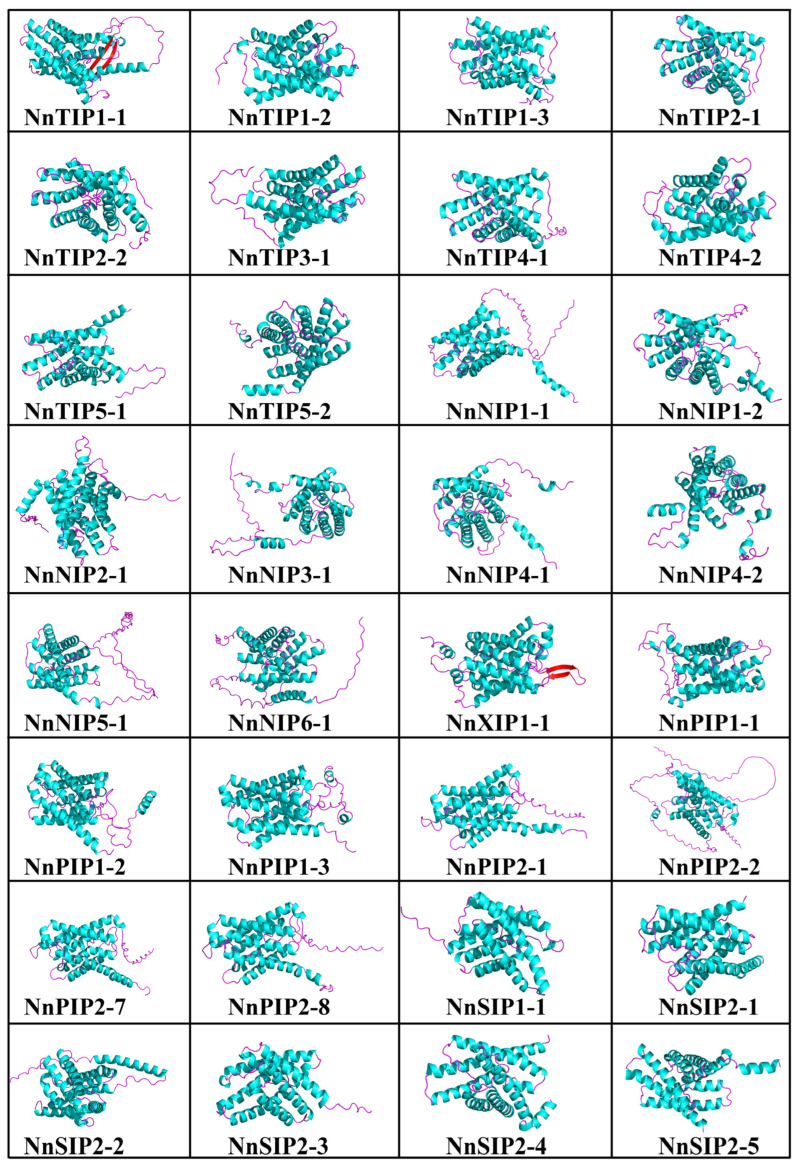
Predicted three-dimensional structures of all 32 NnAQP proteins. Tertiary structures were generated using AlphaFold 3 and visualized with PyMOL. The α-helices are rendered in cyan, random coil/loop regions in purple, and β-strand elements in red (prominently visible in NnTIP1-1 and NnXIP1-1).

The secondary structures of all 32 NnAQP proteins were predicted using the SOPMA online tool, and three-dimensional (3D) tertiary structures were modeled using AlphaFold 3 and visualized with PyMOL. Secondary structure analysis revealed that all NnAQP proteins share a conserved structural pattern predominantly composed of α-helices and random coils. Across all 32 members, the average proportions of α-helices, extended strands, β-turns, and random coils were 35.08%, 18.23%, 1.83%, and 44.87%, respectively. However, notable differences in composition were observed among subfamilies. The SIP subfamily showed the highest average α-helix content (41.06%), with NnSIP2-4 displaying the greatest proportion among all 32 members (45.27%), followed by NnSIP2-3 (43.78%). The TIP subfamily ranked second (average 37.05%), with NnTIP4-2 reaching 42.46% and NnTIP2-2 reaching 41.20%. By contrast, the NIP and PIP subfamilies exhibited lower average α-helix proportions of 31.56% and 31.34%, respectively; NnNIP6-1 (27.51%) and NnPIP2-2 (24.82%) represented the lowest values across the entire family, and both showed correspondingly high random coil proportions. Random coil was the dominant non-helical component across all NnAQPs, while β-turns were consistently the least represented element, accounting for less than 3.5% in all members (Table S3). The predicted 3D tertiary structures of all 32 NnAQP proteins were consistent with the canonical aquaporin fold (Figure 5). As visualized by PyMOL, each protein displayed a compact bundle of cyan transmembrane α-helices interconnected by flexible purple loop regions, forming the characteristic membrane-spanning architecture typical of MIP superfamily members. The overall 3D architectures were highly conserved across all subfamilies, reflecting strong structural constraints imposed by the six-transmembrane helical topology. Notably, NnTIP1-1 and NnXIP1-1 displayed distinct β-strand elements that were not prominently observed in other members, suggesting localized structural variation that may be related to their functional divergence. The candidate protein NnPIP2-7 retained a stable secondary structure configuration consisting of 33.21% α-helices and 47.50% random coils, maintaining the canonical aquaporin fold required for functional transmembrane water channel activity (Figure 5).

### 2.7. Expression Analysis of AQP Genes in Different Tissues of Lotus

**Figure 6:**
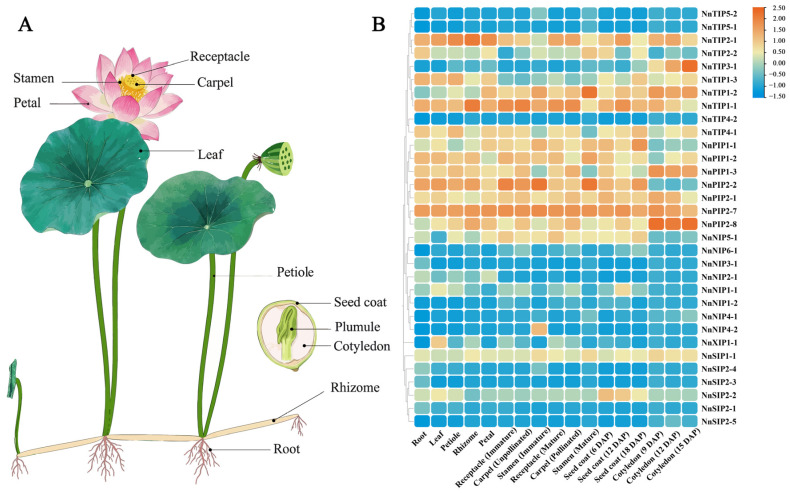
Spatiotemporal expression profiles of *NnAQP* genes across various tissues and developmental stages of lotus. (**A**) Schematic illustration of the lotus plant indicating the anatomical structures sampled. (**B**) Heatmap showing the relative expression levels of *NnAQP* genes across 17 distinct tissue types and developmental stages. Color intensity corresponds to log_2_-transformed FPKM values, with orange-red and blue indicating high and low expression, respectively. DAP, days after pollination.

To investigate the potential function of the 32 *NnAQP* genes during growth and development process of *N. nucifera*, spatiotemporal expression profiles were analyzed using publicly available RNA-seq data [55]. The analyzed samples encompassed diverse plant tissues and developmental stages, including roots, rhizomes, leaves, petioles, petals, immature and mature receptacles and stamens, pollinated and unpollinated carpels, seed coats (6, 12 and 18 days after pollination, DAP), and cotyledons (9, 12 and 15 DAP). Analysis revealed that 30 out of 32 *NnAQP* genes (93.75%) were expressed (FPKM ≥ 1) in at least one of the 17 tissues and development stages examined, while 15 *NnAQP* genes (46.88%) exhibited ubiquitous expression across all tested tissues and stages (FPKM ≥ 1). However, despite their ubiquitous presence, substantial variation in transcript abundance was observed among different tissues for these genes. In contrast, *NnTIP5-1* and *NnTIP4-2* showed minimal or undetectable expression levels in all analyzed samples. Among the ubiquitously expressed members, seven *NnAQP* genes (*NnTIP2-1*, *NnTIP1-1*, *NnTIP1-2*, *NnPIP2-1*, *NnPIP2-7*, *NnPIP2-8*, and *NnSIP1-1*) maintained consistently high transcript abundance (FPKM > 10) in all tested tissues and stages, indicating that these genes may have important roles in plant growth and development. Furthermore, to identify tissue-specific high expression, we analyzed genes with FPKM > 30 in individual tissues. We found that 16, 12, 15, 15, 16, 17 and 12 *NnAQP* genes displayed particularly high transcript abundance in root, rhizome, leaf, petiole, petal, seed coat and cotyledon, respectively; and 13, 11, and 13 genes showed high expression levels (FPKM > 30) in all selected development stages of receptacle, stamen and carpel, respectively (Figure 6B). These results suggest that these genes might be involved in regulating the growth of special tissues. Phylogenetically close *NnAQP* genes exhibited comparable expression patterns. For instance, the subfamily PIPs genes showed relatively high expression level in all tested tissues and stages. In addition, correlation analysis of spatiotemporal expression revealed distinct evolutionary fates among duplicated gene pairs. Some pairs, such as *NnNIP1-1*/*NnNIP1-2* and *NnTIP5-1*/*NnTIP5-2*, shared statistically significant similar expression patterns (Spearman’s ρ=0.504, *p* < 0.05 and ρ=0.498, *p* < 0.05, respectively), suggesting retained functional redundancy. Conversely, other pairs like *NnPIP2-7* and *NnPIP2-8* displayed divergent, non-significant expression correlations (ρ = −0.068, p = 0.794), indicating evolutionary subfunctionalization or neofunctionalization after duplication (Figure 6B, Table S2).

### 2.8. Expression Pattern Analysis of AQP Family Genes in N. nucifera Under Salt Stress Treatments

**Figure 7:**
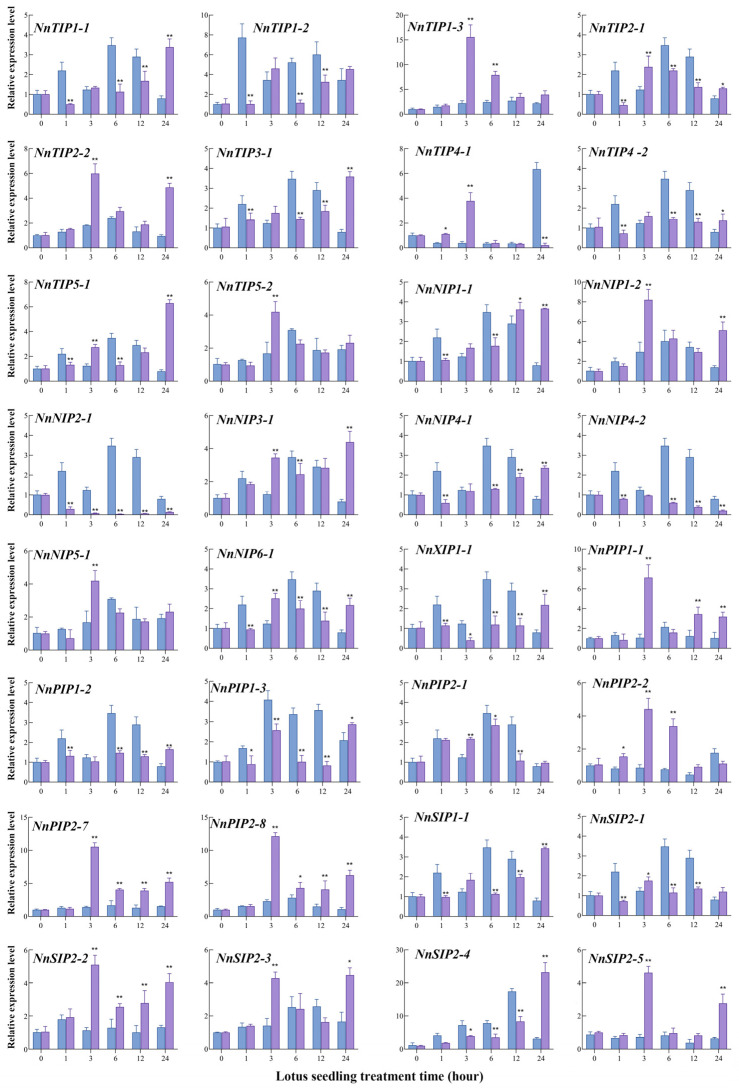
Expression patterns of *NnAQP* genes in response to salt stress. Relative expression levels of all 32 *NnAQP* genes were analyzed by qRT-PCR in the leaves of lotus seedlings at 0, 1, 3, 6, 12, and 24 h after exposure to 200 mM NaCl. The blue bars represent the independent control group, and the purple bars represent the NaCl treatment group at each time point. *NnEF-1α* was used as the internal reference gene. Data are presented as the mean ± SD of three independent biological replicates. Asterisks denote significant differences between the salt-treated group and its independent time-matched control at each specific time point, as determined by Student’s *t*-test (* *p* < 0.05; ** *p* < 0.01). Fold change can be calculated as the ratio of the salt-treated group to its time-matched control.

To investigate the transcriptional responses of *NnAQP* genes under salinity, quantitative real-time PCR (qRT-PCR) analysis was performed on lotus seedlings treated with 200 mM NaCl over a time course of 0, 1, 3, 6, 12, and 24 h. The results revealed that the majority of *NnAQP* genes exhibited significant expression changes, with diverse temporal patterns across subfamilies (Figure 7). In the TIP subfamily, several genes were significantly induced by salt stress. *NnTIP1-3* showed strong up-regulation at 3 h and 6 h, while *NnTIP2-2*, *NnTIP5-1*, and *NnTIP5-2* were markedly induced at specific time points, especially at 3 h or 24 h. In contrast, some TIP genes, such as *NnTIP1-2* and *NnTIP4-2*, showed reduced expression under salt treatment at certain stages, suggesting functional divergence among TIP members. The NIP subfamily displayed diverse expression patterns. *NnNIP1-2*, *NnNIP3-1*, and *NnNIP5-1* were up-regulated under salt stress at specific time points, whereas *NnNIP2-1*, *NnNIP4-1* and *NnNIP4-2* were strongly down-regulated during most treatment periods. These results suggest that NIP genes may have different regulatory roles in response to salt stress. The XIP subfamily contained one detected member, *NnXIP1-1*. Its expression was significantly lower in the salt-treatment group than in the control group at 1, 3, 6, and 12 h, but was up-regulated at 24 h. This pattern indicates that *NnXIP1-1* may be suppressed during the early and middle stages of salt stress, but may participate in the later response to salt-stress adaptation. For the PIP subfamily, several genes responded strongly to salt treatment, including *NnPIP1-1*, *NnPIP2-2*, *NnPIP2-7*, and *NnPIP2-8*. Notably, *NnPIP2-7* exhibited the strongest induction among these genes. At 3 h, the expression level of *NnPIP2-7* was approximately 10-fold higher than that of the control, and it remained consistently higher than the control from 3 to 24 h. In comparison, *NnPIP2-8* was also significantly induced, with an approximately 6-fold increase at 3 h. These results suggest that *NnPIP2-7*, together with *NnPIP2-8*, may be important PIP members involved in the response of *N. nucifera* to salt stress, with *NnPIP2-7* showing a more pronounced response. Additionally, members of the SIP subfamily also displayed salt responsiveness. For example, *NnSIP2-4* exhibited a significant continuous increase in expression, reaching approximately 7-fold higher than the control at 24 h.

In summary, *NnAQP* genes showed diverse expression patterns under salt stress. Among them, *NnPIP2-7* showed the strongest and most sustained induction, with expression approximately 10-fold higher than the control at 3 h and remaining elevated through 24 h, making it the most important candidate gene for further functional study in *N. nucifera*.

### 2.9. Subcellular Localization Analysis of NnAQP Proteins

Predictions of the subcellular localization of NnAQPs using two distinct programs indicated that the majority of NnPIPs, NnNIPs, NnTIPs, and NnXIPs were localized in the plasma membrane. Specifically, most NnTIPs were found to be located in the vacuole, while the remaining ones were likely situated in the plasma membrane (Table 1). The two different subcellular localization prediction tools concurred on the localization of 21 aquaporins (65.6%), predominantly placing them in the plasma membrane and vacuole. For the other 11 aquaporins, Plant-mPLoc predicted a primary localization in the plasma membrane. Conversely, WoLF PSORT predicted several diverse subcellular localizations, including the chloroplast, cytoplasm, and vacuole.

**Figure 8:**
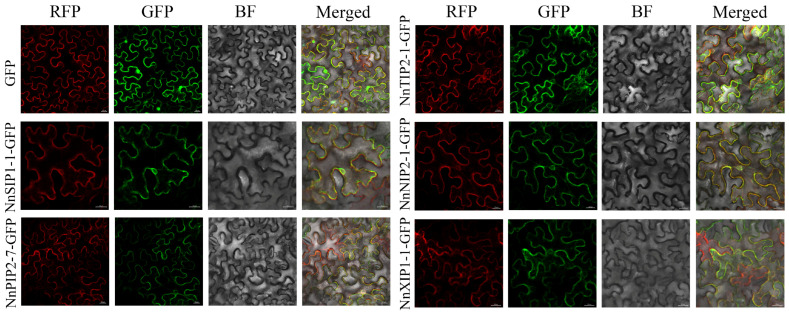
Subcellular localization of five representative NnAQP proteins in *Nicotiana benthamiana* epidermal cells. The coding sequences of *NnPIP2-7*, *NnSIP1-1*, *NnNIP2-1*, *NnTIP2-1*, and *NnXIP1-1* were fused to GFP and transiently co-expressed with the plasma membrane marker AtPIP2a-RFP. The empty GFP vector served as a negative control. From left to right: RFP channel (plasma membrane marker, excited at 561 nm), GFP channel (NnAQP-GFP fusion proteins, excited at 488 nm), bright field (BF), and merged images. GFP, green fluorescent protein; RFP, red fluorescent protein. Bars, 20 µm.

To validate the accuracy of these in silico predictions, five representative NnAQP genes (*NnPIP2-7*, *NnNIP2-1*, *NnSIP1-1*, *NnTIP2-1*, and *NnXIP1-1*) were selected from different subfamilies. Their coding sequences were fused with a green fluorescent protein (GFP) tag and transiently co-expressed with a plasma membrane red fluorescent protein (RFP) marker in *Nicotiana benthamiana* epidermal cells (Figure 8). Confocal microscopy observation revealed that while the control GFP signal was ubiquitously distributed throughout the cytoplasm and nucleus, the green fluorescence of all five NnAQP-GFP fusion proteins co-localized with the PM marker at the cell periphery. This widespread plasma membrane localization indicates their fundamental roles in transmembrane water and solute transport.

Beyond this shared characteristic, distinct subcellular partitioning was observed among the subfamilies. Specifically, NnPIP2-7 and NnNIP2-1 exhibited strict and exclusive plasma membrane localization. In contrast, NnSIP1-1 and NnTIP2-1 displayed additional diffuse fluorescence within the cytoplasm. Furthermore, NnXIP1-1 presented a highly specific reticular fluorescence pattern in the cell interior, confirming its dual localization in both the plasma membrane and the endoplasmic reticulum (ER). These differential subcellular distribution patterns provide robust morphological evidence for the functional diversification and specific organelle compartmentalization of different AQP subfamilies in lotus.

### 2.10. Overexpression of NnPIP2-7 Enhances Salt Tolerance During Seed Germination and Seedling Root Growth in Arabidopsis

**Figure 9:**
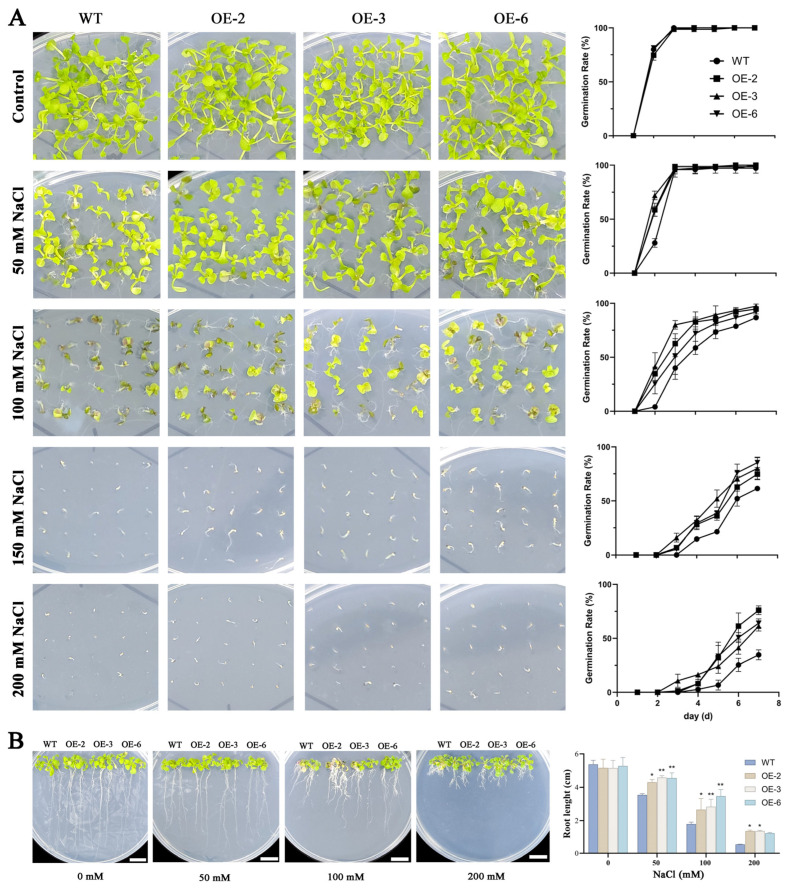
Overexpression of *NnPIP2-7* enhances salt tolerance during seed germination and early seedling root growth in *Arabidopsis*. (**A**) Representative photographs and germination kinetics of wild-type (WT) and three independent *NnPIP2-7* overexpression (OE) lines (OE-2, OE-3, and OE-6) on 1/2 MS medium supplemented with 0, 50, 100, 150, or 200 mM NaCl. Germination rates were recorded daily for 7 days and are presented as mean ± SD of three independent biological replicates. (**B**) Representative photographs and statistical analysis of primary root lengths of WT and OE lines grown on 1/2 MS medium containing 0, 50, 100, or 200 mM NaCl for 7 days. Data represent mean ± SD of three independent biological replicates. Asterisks indicate significant differences between OE line and the WT under the exact same NaCl concentration, as determined by one-way ANOVA followed by Dunnett’s test (* *p* < 0.05; ** *p* < 0.01). Bars, 1 cm.

To assess the salt tolerance of *NnPIP2-7*-overexpressing lines, germination rates and primary root lengths of wild-type (WT) and three independent overexpression (OE) lines (OE-2, OE-3, and OE-6) were evaluated under increasing NaCl concentrations (Figure 9 and Figure S3).

Under control conditions and 50 mM NaCl, all genotypes reached final germination rates of approximately 100% with comparable kinetics, indicating that *NnPIP2-7* overexpression did not affect seed germination under non-stress or mild stress conditions (Figure 9A). As NaCl concentrations increased to 100 and 150 mM, germination rates declined progressively in all genotypes; however, the three OE lines consistently exhibited higher germination rates than WT throughout the monitoring period. The divergence became most pronounced at 200 mM NaCl, at which OE-2, OE-3, and OE-6 reached final germination rates of approximately 65%, 60%, and 80%, respectively, compared with only approximately 35% in WT by day 7 (Figure 9A).

Primary root growth was similarly assessed on medium supplemented with 0, 50, 100, and 200 mM NaCl (Figure 9B). Under control conditions, no significant differences in root length were observed among the four genotypes. Under salt stress, root elongation was progressively inhibited in all lines in a concentration-dependent manner; however, OE lines maintained significantly greater root lengths than WT at 50, 100 and 200 mM NaCl (*p* < 0.05 or *p* < 0.01). Collectively, these results demonstrate that overexpression of *NnPIP2-7* significantly confers enhanced tolerance to salt stress during both seed germination and early seedling establishment in *Arabidopsis*.

### 2.11. NnPIP2-7 Overexpression Alleviates Salt Stress-Induced Damage in Arabidopsis

**Figure 10:**
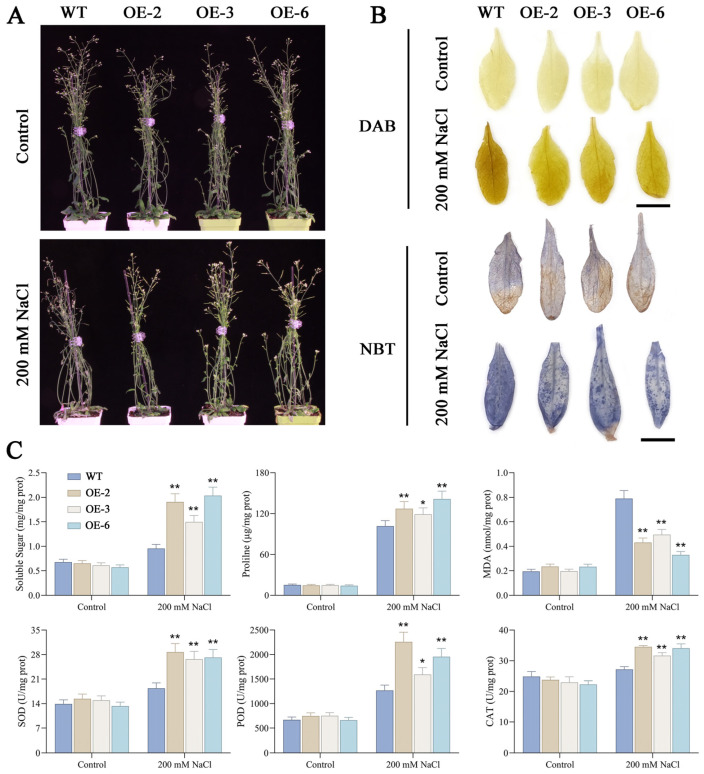
Overexpression of *NnPIP2-7* alleviates salt stress-induced oxidative damage in *Arabidopsis*. (**A**) Representative photographs of four-week-old wild-type (WT) and overexpression (OE) plants before and after 200 mM NaCl treatment for 3 days. (**B**) 3,3′-diaminobenzidine (DAB) and nitroblue tetrazolium (NBT) staining of leaves from WT and OE plants under control and salt stress conditions, indicating the accumulation of H_2_O_2_ and O_2_^−^, respectively. Bars, 1 cm. (**C**) Contents of soluble sugar, proline, and malondialdehyde (MDA), and activities of superoxide dismutase (SOD), peroxidase (POD), and catalase (CAT) in the leaves of WT and OE lines under control and 200 mM NaCl treatments. Data are presented as the mean ± SD of three independent biological replicates. Asterisks indicate significant differences between the OE line and the WT under the same specific treatment condition (control or 200 mM NaCl), as determined by one-way ANOVA followed by Dunnett’s test (* *p* < 0.05; ** *p* < 0.01).

Four-week-old WT and transgenic plants were treated with 200 mM NaCl for 3 days (Figure 10A). Under control conditions, no visible phenotypic differences were observed among the four genotypes. Following salt treatment, WT plants exhibited pronounced leaf wilting and chlorosis, whereas OE lines maintained a more upright growth habit with markedly less leaf damage. To visually assess the accumulation of ROS triggered by salt stress, histochemical staining with 3,3′-diaminobenzidine (DAB) and nitroblue tetrazolium (NBT) was performed to detect H_2_O_2_ and O_2_^−^, respectively. Under control conditions, both WT and transgenic leaves exhibited minimal background staining. However, following the 200 mM NaCl treatment, WT leaves displayed deep brown and dark blue staining, indicating extensive ROS accumulation. In contrast, the OE lines showed significantly lighter staining intensities (Figure 10B), demonstrating that *NnPIP2-7* overexpression effectively restricts excessive ROS accumulation.

To investigate the physiological basis of this tolerance, soluble sugar, proline, malondialdehyde (MDA) contents, and the activities of superoxide dismutase (SOD), peroxidase (POD), and catalase (CAT) were measured under control and salt stress conditions (Figure 10C). Under control conditions, no significant differences were detected among all genotypes. Upon salt treatment, soluble sugar and proline contents were significantly higher in OE lines than in WT (*p* < 0.01), while MDA content was significantly lower (*p* < 0.01), indicating enhanced osmotic adjustment and reduced membrane oxidative damage in transgenic plants. Consistently, SOD, POD, and CAT activities were all significantly elevated in OE lines relative to WT (*p* < 0.05 or *p* < 0.01). These results collectively indicate that *NnPIP2-7* overexpression improves salt tolerance in *Arabidopsis* by enhancing osmolyte accumulation and antioxidant capacity while reducing oxidative damage.

## 3. Discussion

The genome-wide identification of 32 *NnAQP* members in *Nelumbo nucifera* provides a fundamental genomic framework for understanding water and solute transport in this basal eudicot. The family size is highly comparable to that of other diploid model species, such as *Arabidopsis* with 35 members [28] and *Oryza sativa* with 33 members [37], but significantly smaller than paleopolyploid species like *Gossypium hirsutum* with 68 members [38]. From an evolutionary standpoint, the *Nelumbo nucifera* genome experienced a whole-genome duplication (WGD) event approximately 65 Mya, followed by an extensive diploidization process [56]. Notably, the seven pairs of segmentally duplicated genes identified in this study exhibited estimated divergence times ranging from 39.43 to 53.74 Mya. Rather than strictly coinciding with the WGD event itself, this chronological window corresponds perfectly to the subsequent prolonged phase of diploidization. This chronological alignment strongly implies that the 32 extant *NnAQP* members are the retained remnants of a massive gene loss and rapid diploidization process following paleopolyploidy. Consequently, this streamlined genomic distribution perfectly aligns with the diploid nature of the lotus genome and its stable aquatic evolutionary history [56,57].

This post-WGD gene retention, however, was not uniform across subfamilies, resulting in an evolutionarily unbalanced distribution of *NnAQP* members. For instance, the XIP subfamily was reduced to the single member *NnXIP1-1* in lotus. This pattern is consistent with the biased gene loss model during diploidization [58,59], wherein genes with overlapping or dispensable functions are preferentially eliminated during genome fractionation [60]. This is also concordant with the broader phylogenetic pattern in which XIPs are absent from monocots and show markedly reduced representation in many angiosperm lineages [17]. In addition, this evolutionary sorting process also manifested as variations in selective pressures among the retained paralogs. Within the NIP subfamily, *NnNIP3-1* and *NnNIP5-1* exhibited secondary exon loss, and the duplicated pair *NnNIP4-1*/*NnNIP4-2* presented a relatively elevated *Ka*/*Ks* ratio of 0.330, hinting at a localized relaxation of purifying selection or accelerated subfunctionalization. Considering the long-term aquatic adaptation and relatively stable evolutionary history of *Nelumbo*, the contraction of specific AQP lineages alongside the preservation of others likely reflects a deep ecological specialization of water transport systems in aquatic environments.

Despite these lineage-specific variations, the baseline evolutionary trajectory of the entire lotus AQP family remains strictly governed by intense purifying selection. This is evidenced by the *Ka*/*Ks* ratios of all segmentally duplicated pairs being strictly less than 1, a stringent overarching pressure essential to maintain the functional integrity of water channels and prevent mutational divergence [15]. Such rigid evolutionary constraints are directly manifested at the structural level. According to the protein secondary structure prediction in Supplementary Table S3, all NnAQP subfamilies retained a highly conserved structural architecture predominantly composed of transmembrane α-helices which averaged 31.34% to 41.06%, alongside random coils, consistent with the canonical topology of aquaporins in which α-helical domains form the membrane-spanning pore while flexible loop/coiled regions contribute to conformational dynamics associated with transport activity [23]. For instance, the core candidate protein NnPIP2-7 retains a stable structural configuration consisting of 33.21% α-helices and 47.50% random coils, perfectly safeguarding its channel integrity under environmental perturbations.

These structural constraints are further underscored by the universal preservation of motifs 2, 3, and 4 across almost all NnAQP members. These conserved elements correspond to the functionally indispensable NPA motifs and the aromatic/arginine (ar/R) selectivity filter [8,24]. Specific structural variations eventually emerged under lineage-specific subfunctionalization, including the reduced motif complement in the SIP subfamily and the secondary exon loss in *NnNIP3-1* and *NnNIP5-1.* Despite these features being documented in other angiosperms [28,61,62], the core gating topology of lotus AQPs remains highly conserved. Collectively, this indicates that early subfunctionalization successfully accommodated distinct subcellular transport requirements while preserving essential transmembrane transport capacities.

Beyond genomic structural conservation, the functional divergence of *NnAQPs* is prominently manifested in their transcriptional reprogramming under environmental constraints. In response to fluctuating external osmotic pressures, plants must rapidly adjust their hydraulic conductivity by modulating AQP transcription [20,63]. The diverse temporal expression profiles of *NnAQP* subfamilies under salt stress reflect a highly compartmentalized regulatory network. For instance, the significant down-regulation of specific NIP members, such as *NnNIP2-1*, *NnNIP4-1*, likely acts as an active defense strategy. Given that specific NIP subgroups are permeable to potentially toxic metalloids or excess ions, their transcriptional suppression under saline conditions prevents the continuous influx of harmful solutes when cellular ion homeostasis is already challenged [24,64]. In stark contrast to the suppression of NIPs, the pronounced and rapid induction of multiple PIP members points to their dominant role in osmotic adjustment. This was particularly evident for *NnPIP2-7*, which exhibited an approximately 10-fold up-regulation at 3 h. PIP2 proteins, primarily localized to the plasma membrane, intrinsically possess higher water channel activity than other subfamilies [63,65,66]. The intense transcriptional activation of *NnPIP2-7* suggests that lotus actively recruits these specific channels to facilitate rapid water uptake, counteracting the hypertonic external environment.

This rapid transcriptional reprogramming is mechanistically supported by the enrichment of stress-responsive cis-elements, particularly ABA-responsive elements (ABREs), identified in their promoters. In plants, ABREs serve as quintessential molecular switches for abiotic stress adaptation, translating stress-induced abscisic acid (ABA) accumulation into rapid, genome-wide transcriptional reprogramming [1]. Recent molecular evidence within the aquaporin field explicitly corroborates this regulatory mechanism. For instance, under environmental stress, ABRE-binding factors (ABFs) have been proven to directly interact with the ABRE motifs within the PIP2 aquaporin promoter, acting as primary transcriptional activators to positively drive its rapid expression [67]. Consequently, the high frequency of ABREs implies that *NnAQP* expression is tightly integrated into ABA-dependent signaling cascades, allowing the plant to translate early osmotic stress signals into rapid adjustments in membrane water permeability.

*NnPIP2-7* was prioritized for functional validation over its paralog *NnPIP2-8* on the basis of its markedly stronger and more sustained transcriptional induction under salt stress. Specifically *NnPIP2-7* showed a 10-fold increase compared to a 6-fold increase for *NnPIP2-8* at 3 h, which is consistent with the subfunctionalization of this duplicated gene pair inferred from the genomic analysis. The physiological significance of *NnPIP2-7* in mediating this stress response was further validated through its heterologous overexpression in *Arabidopsis*. Under varying NaCl concentrations, transgenic lines consistently exhibited superior germination rates and primary root elongation compared to wild-type plants. Sustained root elongation under saline conditions strictly depends on the maintenance of cell turgor through continuous water influx [5], a biophysical process where the plasma membrane-localized NnPIP2-7 acts as a crucial molecular gatekeeper.

Moreover, biochemical parameter analysis provided valuable insights into the physiological responses associated with the overexpression of this channel protein. Salt stress inevitably induces secondary oxidative stress, which leads to the accumulation of ROS and consequent lipid peroxidation [44]. The significant reduction in MDA content in overexpression lines, coupled with elevated activities of SOD, POD, and CAT, indicates a strong correlation between *NnPIP2-7* expression and the mitigation of oxidative damage to cellular membranes. Recent evidence suggests that specific AQPs can facilitate the transmembrane diffusion of H_2_O_2_, enabling its redistribution to cellular compartments where antioxidant enzymes are concentrated [41,44]. While our direct biochemical evidence shows enhanced antioxidant capacity, whether *NnPIP2-7* directly transports ROS or indirectly alleviates oxidative stress through improved water status remains a plausible interpretation requiring further molecular validation. Furthermore, the significantly higher accumulation of proline and soluble sugars in transgenic lines is consistent with improved osmotic adjustment [5,45]. These accumulated osmolytes likely help lower the intracellular osmotic potential to sustain water uptake, functionally complementing the water channel activity of *NnPIP2-7*. Collectively, while our direct evidence confirms the physiological enhancement of salt tolerance, these findings suggest that the protective role of *NnPIP2-7* extends beyond acting solely as a passive water pore. It is plausible that its activity synergistically facilitates the preservation of osmotic balance and the mitigation of oxidative stress, though the complete regulatory network warrants future investigation.

It is worth noting that while the heterologous expression in *Arabidopsis* provides robust foundational evidence for the salt-tolerance function of *NnPIP2-7*, this model system has inherent limitations. Lotus, as an aquatic plant, possesses unique physiological and anatomical adaptations, such as well-developed aerenchyma and distinct root hydraulic architectures, which differ fundamentally from terrestrial dicots. The precise in *planta* regulatory network and transport dynamics of *NnPIP2-7* might involve lotus-specific interacting partners or cell-type-specific localization that cannot be fully recapitulated in *Arabidopsis*. Future investigations utilizing stable genetic transformation or virus-induced gene silencing (VIGS) directly within *Nelumbo nucifera* will be essential to elucidate its authentic physiological roles in the context of aquatic environmental adaptations.

Finally, given the close evolutionary relationship between *N. nucifera* and *N. lutea*, we anticipate a high degree of structural and functional conservation within the AQP gene family between these two species. Our synteny analysis indicated that most *NnAQP* genes possess orthologous counterparts in the *N. lutea* genome (Figure 4), suggesting that the genomic architecture of this family is likely conserved. Specifically, regarding the function of *NnPIP2-7* in salt tolerance, it is highly probable that its ortholog in *N. lutea* serves a similar regulatory role in osmotic adjustment. Nevertheless, *N. nucifera* and *N. lutea* exhibit distinct ecological adaptations and environmental responses. Therefore, while the core mechanism of *PIP2-7*-mediated salt tolerance is likely shared, subtle variations in transcriptional regulation or protein activity may exist to accommodate the specific environmental resilience of each species. Future comparative studies between these two *Nelumbo* species will be essential to precisely define the extent of this functional conservation and potential species-specific adaptations.

## 4. Materials and Methods

### 4.1. Genome-Wide Sequence Retrieval and Identification

The AQP protein sequences of *Arabidopsis thaliana* and *Oryza sativa* were retrieved from the TAIR database (https://www.arabidopsis.org/, accessed on 1 August 2024) and the Rice Genome Annotation Project (http://rice.plantbiology.msu.edu/, accessed on 9 August 2024), respectively. This combination of monocot and dicot models provides robust, broad-spectrum reference sequences for the genome-wide homology-based search. Given the absence of XIP subfamily members in both *Arabidopsis* and rice, XIP sequences from *Lotus japonicus* and *Solanum lycopersicum* were additionally obtained to provide essential phylogenetic coverage, thereby ensuring the comprehensive identification of all potential NnAQP homologs in the *Nelumbo nucifera* genome. These sequences were subsequently employed as queries to search for candidate AQP protein sequences in the *Nelumbo* genome database (http://nelumbo.cngb.org/nelumbo/home, accessed on 9 August 2024) using the online BLASTP tool with an E-value cutoff of 1 × 10^−5^ [68]. To confirm the accuracy of AQP gene family identification in lotus, the conserved domain profile of the MIP family (PF00230) was retrieved from the Pfam database (http://pfam.xfam.org/, accessed on 9 August 2024) and subsequently used to identify candidate lotus AQP members through Hidden Markov Model (HMM) analysis on the HMMER web server (https://www.ebi.ac.uk/Tools/hmmer/, accessed on 10 August 2024). For genes with multiple alternative splicing variants, only the primary (longest) transcript was retained. Redundant sequences identical in their coding regions were manually removed.

The transmembrane helical domains of all candidate AQP protein sequences were further verified using the DeepTMHMM−1.0 server (https://services.healthtech.dtu.dk/services/DeepTMHMM-1.0/, accessed on 18 January 2025). The physicochemical properties of these AQP proteins were predicted using ExPASy (https://www.expasy.org, accessed on 1 May 2025) [69], and their subcellular localization was predicted using Plant-mPLoc 2.0 tool (http://www.csbio.sjtu.edu.cn/bioinf/plant-multi/, accessed on 18 May 2025) [70] and WoLF PSORT (https://wolfpsort.hgc.jp/, accessed on 18 May 2025) [71]. Secondary structures of the AQP proteins were predicted using the SOPMA online tool (https://npsa-prabi.ibcp.fr/cgi-bin/npsa_automat.pl?page=npsa_sopma.html, accessed on 20 May 2025), and the proportions of α-helices, extended strands, β-turns, and random coils were analyzed for each protein. Three-dimensional (3D) structures were predicted using AlphaFold 3 (https://alphafold.ebi.ac.uk/, accessed on 20 May 2025) and visualized using PyMOL (v3.0.3).

### 4.2. Sequence Alignment and Phylogenetic Tree Construction

Multiple sequence alignment of AQP protein sequences from *N. nucifera*, *A. thaliana*, *O. sativa*, *L. japonicus*, and *S. lycopersicum* was performed using ClustalW (https://www.genome.jp/tools-bin/clustalw, accessed on 15 May 2025). A phylogenetic tree of the AQP family was subsequently constructed using the NJ method implemented in MEGA 11 [72], with 1000 bootstrap replicates. The resulting phylogenetic tree was visualized and edited using ChiPlot (https://www.chiplot.online/, accessed on 15 May 2025).

### 4.3. Analysis of the Conserved Motifs, Domains Architecture, Gene Structure and Promoter Elements of Lotus AQP Genes

AQP protein sequences were retrieved from the *Nelumbo* genome database (http://nelumbo.cngb.org/nelumbo/, accessed on 15 May 2025). Conserved motifs were identified using MEME Suite 5.5.9 [73], with a motif-level E-value threshold set to 1 × 10^−5^ (https://meme-suite.org/meme/tools/meme, accessed on 11 April 2025). Conserved domain architectures were analyzed with NCBI Conserved Domain Database (CDD) using default parameters (https://www.ncbi.nlm.nih.gov/cdd, accessed on 11 April 2025). Exon–intron structures and UTRs were annotated in TBtools (v2.476) based on the genome GFF3 annotation file. The conserved motifs, domains, and gene structures were integrated and visualized using the Gene Structure View (Advanced) module of TBtools [74]. To investigate the cis-regulatory elements within the promoter regions, the 2000 bp sequences upstream of the start codon of each AQP gene were extracted and submitted to the PlantCARE database (http://bioinformatics.psb.ugent.be/webtools/plantcare/html/; accessed on 12 April 2025) for cis-element prediction [75].

### 4.4. Collinearity Analysis of Lotus AQP Family Genes

The genome and annotation files of *Arabidopsis thaliana* and *Oryza sativa* were downloaded from Ensembl Plants (https://plants.ensembl.org/index.html, accessed on 15 April 2025), while the corresponding files for lotus were obtained from the *Nelumbo* genome database (http://nelumbo.cngb.org/nelumbo/, accessed on 15 April 2025). Inter- and intra-species collinearity analyses were performed using the One-Step MCScanX module integrated with TBtools, and the results were visualized using the Advanced Circos module [73]. Non-synonymous (Ka) and synonymous (Ks) substitution rates of AQP gene pairs were calculated using the Simple Ka/Ks Calculator (NG method) implemented in TBtools. Divergence times were estimated using the formula T = Ks/(2 × 6.1 × 10^−9^) × 10^−6^, where T is expressed in million years ago (Mya) [76].

### 4.5. Expression Analysis of NnAQP Genes Across Different Tissues of Nelumbo nucifera

Fragments per kilobase of transcript per million mapped reads (FPKM) expression data for *NnAQP* genes across 17 tissues and developmental stages were obtained from the *Nelumbo* genome database [55], including root, rhizome, leaf, petiole, petal, immature receptacle, mature receptacle, immature stamen, mature stamen, pollinated carpel, unpollinated carpel, seed coat at 6, 12, and 18 days after pollination, as well as cotyledon at 9, 12, and 15 days after pollination. A heatmap was generated using TBtools with color intensity corresponding to log_2_-transformed FPKM values. For the present analysis, an empirical threshold of FPKM ≥ 1 was selected to define actively expressed genes. This criterion was strategically adopted to effectively distinguish true biological transcription from technical background noise, a standard methodological approach recently validated in other genome-wide transcriptomic analyses within *Nelumbo nucifera* [52]. Furthermore, to evaluate the co-expression patterns and potential functional redundancy of segmentally duplicated gene pairs, Spearman’s rank correlation coefficients $\left(\rho \right)$ were calculated based on their FPKM profiles across all tested tissues.

### 4.6. Plant Materials and Treatments

Seeds of *Nelumbo nucifera* cultivar ‘China Antique’ were surface-scarified at the blunt end and germinated in distilled water for one week. Uniformly developed seedlings were then transferred to 1/2 Hoagland nutrient solution and grown hydroponically for two weeks under controlled conditions at Jinhua Academy of Agricultural Sciences, maintaining an average day/night temperature of 30 °C/23 °C under natural light conditions. Three-week-old seedlings at the two-leaf stage were divided into parallel groups for the time-course experiment. The salt-stress group was subjected to 200 mM NaCl dissolved in the nutrient solution. This specific concentration was determined by referencing previous studies that utilized 250 mM NaCl on older, four-leaf-stage lotus seedlings [51], and adjusting it based on our preliminary dose–response assessments. The control group was continuously maintained in the standard 1/2 Hoagland nutrient solution without the addition of NaCl. Leaf samples from both the salt-treated group and the control group were collected simultaneously at 0, 1, 3, 6, 12, and 24 h after treatment. Crucially, this experimental design ensured that each individual treatment time point had its own independent control group rather than sharing a single control at zero hours, thereby effectively accounting for any potential circadian or developmental fluctuations in baseline gene expression. The experiment was performed with three biological and three technical replicates. All collected samples were immediately frozen in liquid nitrogen, and stored at −80 °C until further analysis.

For germination assays, surface-sterilized seeds of wild-type (WT) and transgenic *Arabidopsis* lines were sown on 1/2 Murashige and Skoog (MS) medium supplemented with 0 (control), 50, 100, 150, or 200 mM NaCl. Seeds were stratified at 4 °C in the dark for 3 days, and then transferred to a growth chamber maintained at 22 °C with a 16 h light/8 h dark photoperiod. Germination (defined as radicle emergence > 1 mm) rates were recorded after 7 days. For root length measurements, sterilized seeds were germinated on 1/2 MS medium for 5 days, after which seedlings were transferred to medium containing 0 (control), 50, 100 or 200 mM NaCl and grown for an additional 7 days before root length was determined using Image J software (v1.54g). For whole-plant salt stress assays, four-week-old seedlings of WT and T3 homozygous transgenic lines overexpressing *NnPIP2-7* were treated with 200 mM NaCl. Concurrently, a parallel set of plants of all genotypes was maintained under normal watering conditions to serve as the unstressed control group. Phenotypic responses and physiological parameters for both the treated and control groups were recorded at 3 days post-treatment.

### 4.7. Total RNA Extraction and qRT-PCR Analysis

Total RNA was extracted from lotus leaves using Trizol reagent (Invitrogen, Carlsbad, CA, USA) following the manufacturer’s instructions. First-strand cDNA was synthesized from 1 µg of total RNA using the M5 Super Plus qPCR RT Kit with gDNA Remover (Mei5bio, Beijing, China) to eliminate genomic DNA contamination. qRT-PCR analyses were subsequently performed on a Bio-Rad CFX96 Real-Time PCR Detection System (Bio-Rad, Hercules, CA, USA) using SYBR Green Real-Time PCR Master Mix (TaKaRa, Dalian, China). The *NnEF-1α* gene was used as the internal reference for normalization [77]. Relative expression levels were calculated using the 2^−∆∆Ct^ method [78], with the transcript abundance of each salt-treated sample being strictly normalized against its corresponding time-matched independent control to accurately reflect the stress-induced fold changes. All assays were conducted with three independent biological replicates, each with three technical replicates. Primer sequences used for qRT-PCR are listed in Supplementary Table S4.

### 4.8. Transient Expression and Confocal Microscopy for Subcellular Localization

The coding sequences of selected *NnAQP* genes were amplified by PCR and cloned into the pRI101-EGFP vector. The recombinant constructs were transferred into *Agrobacterium tumefaciens* GV3101 via the heat-shock method and subsequently expressed transiently in the leaves of four-week-old *Nicotiana benthamiana* plants by agroinfiltration. An empty pRI101-EGFP vector was also infiltrated under identical conditions to serve as a negative control. The plasma membrane marker AtPIP2a-RFP was co-expressed as a subcellular reference [79]. Fluorescence signals were detected using a confocal laser-scanning microscope (LSM 800, Carl Zeiss, Oberkochen, Germany). GFP was excited at 488 nm and detected at 500–550 nm, while RFP was excited at 561 nm and detected at 580–630 nm. Primer sequences used for gene cloning are listed in Supplementary Table S4.

### 4.9. Genetic Transformation and Screening of Transgenic Arabidopsis Lines

The coding sequence of *NnPIP2-7* was cloned into the pCAMBIA3301 vector and introduced into *Arabidopsis thaliana* (ecotype Columbia) via *Agrobacterium tumefaciens*-mediated floral dip transformation [80]. Transgenic T1 plants were screened on MS medium supplemented with 15 mg/L glufosinate-ammonium. Overexpression of *NnPIP2-7* in selected lines was confirmed by qRT-PCR (using *AtTublin* as the internal reference gene), and three independent T3 homozygous transgenic lines were selected for subsequent analysis. Primer sequences used for vector construction are listed in Supplementary Table S4.

### 4.10. Histochemical Staining and Physiological Parameter Analysis

For DAB and NBT staining, leaf samples were immersed in 1 mg/mL DAB (50 mM Tris-HCl, pH = 5.5) and 1 mg/mL NBT (0.2 M PBS pH = 7.8) for 12 h, respectively, and then decolorized in 95% (*v*/*v*) ethanol until the chlorophyll was fully removed. Images were acquired using a Canon 80D (Canon, Tokyo, Japan) camera. For physiological parameter measurements, approximately 100 mg of leaf tissues was used to determine the contents of proline, soluble sugar, and MDA, as well as the activities of CAT, SOD, and POD following established protocols [81].

### 4.11. Statistical Analysis

All statistical analyses were performed using GraphPad Prism 8.0.2 (GraphPad, La Jolla, CA, USA). Prior to parametric testing, all data were evaluated for normal distribution using the Shapiro–Wilk test and for homogeneity of variances using Levene’s test. Student’s *t*-test was applied for pairwise comparisons between the treatment and corresponding control groups. For multiple group comparisons among the wild-type and transgenic lines, a one-way analysis of variance (ANOVA) followed by Dunnett’s post hoc test was utilized. Statistical significance was defined as *p* < 0.05.

## 5. Conclusions

The aquaporin gene family in *Nelumbo nucifera* undergoes evolutionary expansion primarily driven by segmental duplication and is maintained under stringent purifying selection. Within this family, the plasma membrane-localized *NnPIP2-7* functions as a core molecular regulator in the rapid response to salt stress. *NnPIP2-7* confers enhanced plant salt tolerance by synergistically facilitating intracellular osmotic adjustment and mitigating salt-induced oxidative damage, thereby sustaining cellular homeostasis under high-salinity environments.

## Figures and Tables

**Table 1:** Characteristics of the 32 AQP genes identified from the lotus genome.

Gene Name	Gene ID	Protein Length (aa)	Molecular Weight (kDa)	Theoretical pI	Grand Average of Hydropathicity	TMDs	Subcellular Localization Prediction	
							WoLF PSORT	Plant-mPLoc
NnNIP1-1	Nn3g21048.3	287	30.74	8.57	0.48	6	Plas	Plas
NnNIP1-2	Nn6g34451.1	287	30.89	8.79	0.43	6	Plas	Plas
NnNIP2-1	Nn4g23170.1	296	31.61	7.88	0.233	6	Plas	Plas
NnNIP3-1	Nn5g30734.1	297	31.41	9.03	0.518	6	Vacu	Plas
NnNIP4-1	Nn4g24146.1	275	29.24	8.91	0.694	6	Plas	Plas
NnNIP4-2	Nn3g18222.1	282	30.12	7.66	0.692	6	Plas	Plas
NnNIP5-1	Nn7g37195.3	300	31.25	8.87	0.395	6	Plas	Plas
NnNIP6-1	Nn8g39814.1	338	35.2	8.78	0.538	6	Vacu	Plas
NnPIP1-1	Nn2g11416.2	286	30.94	8.37	0.36	6	Plas	Plas
NnPIP1-2	Nn2g13650.2	286	30.72	8.82	0.366	6	Plas	Plas
NnPIP1-3	Nn1g01548.2	286	30.94	8.86	0.382	6	Plas	Plas
NnPIP2-1	Nn2g13095.1	287	30.72	8.81	0.475	6	Plas	Plas
NnPIP2-2	Nn2g10794.1	407	44.18	9.3	0.221	6	Plas	Plas
NnPIP2-7	Nn5g30807.1	280	29.68	9.03	0.481	6	Plas	Plas
NnPIP2-8	Nn6g31954.4	280	29.86	9.23	0.45	6	Plas	Plas
NnSIP1-1	Nn4g25032.3	237	25.25	9.7	0.788	6	Vacu	Plas/Vac
NnSIP2-1	Nn7g37157.1	238	26.96	9.88	0.435	6	Chlo	Plas/Vac
NnSIP2-2	Nn7g37160.1	293	32.27	9.11	0.337	6	Extr	Plas
NnSIP2-3	Nn7g37156.1	233	25.22	8.9	0.49	6	Nucl	Plas/Vac
NnSIP2-4	Nn7g37155.1	243	27.42	5.93	0.484	6	Cyto	Plas
NnSIP2-5	Nn7g37159.1	239	26.58	9.39	0.331	6	Plas	Plas
NnTIP1-1	Nn6g35224.10	252	26	5.79	0.717	5	Plas	Vac
NnTIP1-2	Nn2g13853.1	251	25.97	6.27	0.734	6	Plas	Vac
NnTIP1-3	Nn2g10491.1	251	25.87	5.35	0.8	6	Plas	Vac
NnTIP2-1	Nn2g11641.1	249	25.12	6.01	0.951	6	Vacu	Vac
NnTIP2-2	Nn6g33920.1	250	25.39	5.35	0.906	6	Vacu	Vac
NnTIP3-1	Nn3g18522.2	259	26.79	6.75	0.682	6	Plas	Vac
NnTIP4-1	Nn3g18154.1	248	25.74	5.8	0.827	6	Plas	Vac
NnTIP4-2	Nn2g11645.1	252	26.23	7.69	1.012	6	Vacu	Vac
NnTIP5-1	Nn6g33919.1	257	26.25	6.8	0.779	6	Plas/E.R.	Plas/Vac
NnTIP5-2	Nn1g00962.1	257	26.24	5.82	0.809	6	Plas	Plas/Vac
NnXIP1-1	Nn1g05881.1	306	32.98	7.68	0.581	6	Plas	Plas

Note: aa: amino acids; kDa: kilodaltons; pI: isoelectric point; TMDs: transmembrane domains. Subcellular localization abbreviations: Plas: plasma membrane; Vac/Vacu: vacuole; Chlo: chloroplast; Extr: extracellular; Nucl: nucleus; Cyto: cytoplasm; E.R.: endoplasmic reticulum.

## Data Availability

The original contributions presented in this study are included in the article/Supplementary Materials. Further inquiries can be directed to the corresponding authors.

## Supplementary Materials

The following supporting information can be downloaded at: https://www.mdpi.com/article/10.3390/plants15142186/s1, Figure S1: Chromosomal distribution of the 32 *NnAQP* genes in the *Nelumbo nucifera* genome; Figure S2: Multiple sequence alignment of 32 NnAQP proteins; Figure S3: Identification and screening of *NnPIP2-7* overexpressing transgenic *Arabidopsis* lines; Table S1: Amino acid compositions of the NPA motifs, Ar/R selectivity filters, and Froger’s positions of NnAQPs; Table S2: Tandemly and segmentally duplicated *NnAQP* gene pairs; Table S3: Protein secondary structure of NnAQPs in *N. nucifera*; Table S4: Sequences of primer used in this study.
