# Supplementary material for: Genome-Wide Analysis of the Aquaporin Gene Family Reveals the Role of NnPIP2-7 in Conferring Salt Tolerance in Lotus (Nelumbo nucifera Gaertn.)
Source: Plants (Basel). 2026 Jul 16;15(14):2186. doi: 10.3390/plants15142186 (PMC13414738; doi:10.3390/plants15142186)
Supplement: Supplementary file 1 [file plants-15-02186-s001.zip › Supplementary Figure S1-S3.pdf]

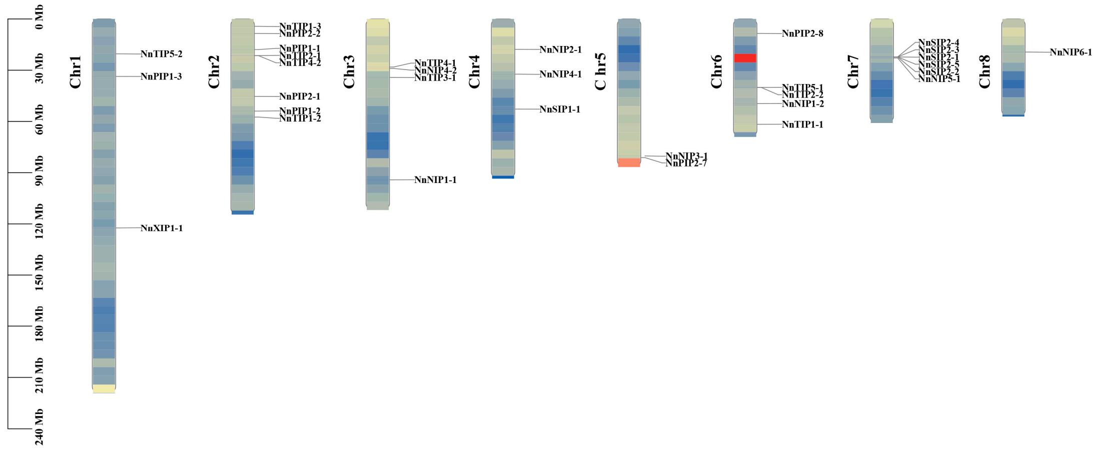

Figure S1 Chromosomal distribution of the 32 NnAQP genes in the *Nelumbo nucifera* genome. The vertical bars represent the eight lotus chromosomes (Chr1–Chr8). The 32 identified NnAQP genes are mapped to their corresponding physical positions, with gene names indicated on the right side of each chromosome. The scale bar on the left represents the chromosomal length in megabases (Mb).

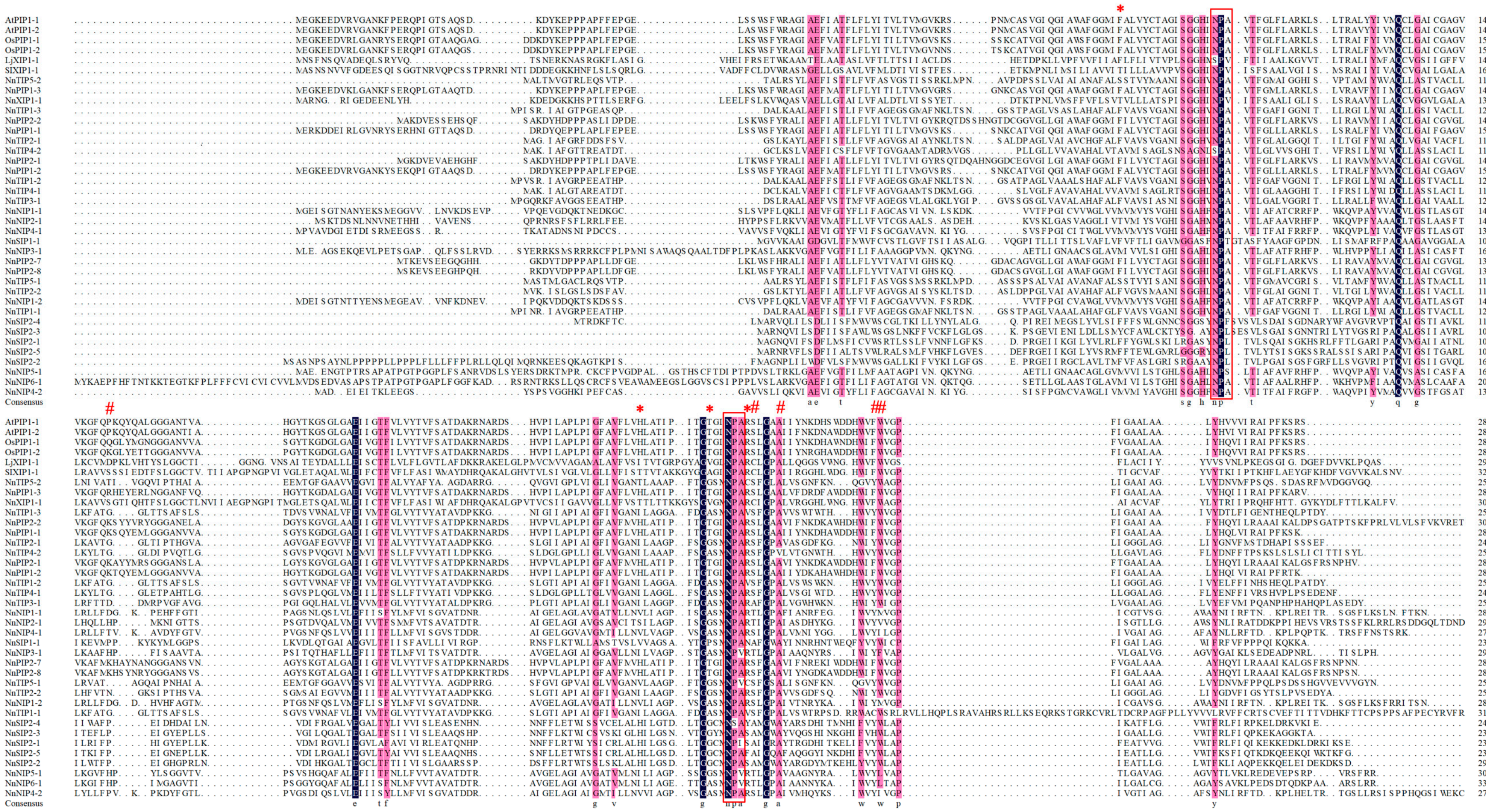

Figure S2 Multiple sequence alignment of 32 NnAQP proteins. The red boxes highlight the two highly conserved NPA motifs; red asterisks (*) indicate the four residues of the Ar/R selectivity filter; and hash symbols (#) denote the five Froger's positions. The consensus sequence is shown at the bottom.

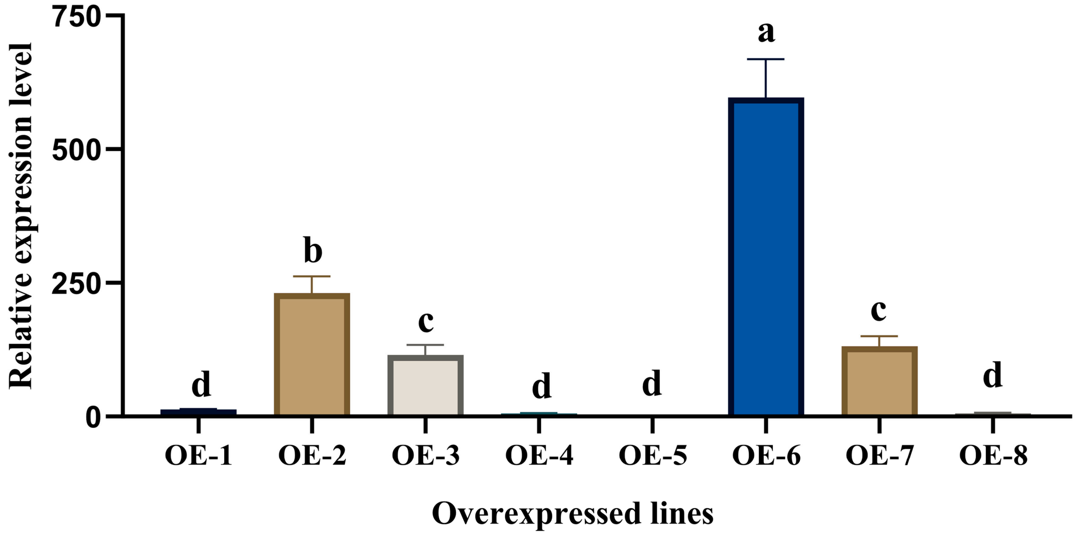

Figure S3 Identification and screening of *NnPIP2-7* overexpressing transgenic *Arabidopsis* lines. Relative expression levels of *NnPIP2-7* in eight independent transgenic lines (OE-1 to OE-8) were determined by qRT-PCR. Based on the expression abundance, lines OE-2, OE-3, and OE-6 were selected for subsequent stress tolerance assays. Data are presented as the mean ± SD of three independent biological replicates. Different lowercase letters above the bars indicate statistically significant differences ($p < 0.05$) among the lines, as determined by one-way ANOVA followed by Tukey's test.
